# The Rheological Enhancement of an Internal Olefin Sulphonate Surfactant upon Interactions with Cationic Surfactants by Micellization Changes

**DOI:** 10.3390/ma18061270

**Published:** 2025-03-13

**Authors:** Ana María Lozada, María Isabel Sandoval, Ronald Mercado

**Affiliations:** 1 Grupo de Investigación en Fenómenos Interfaciales, Reología y Simulación de Transporte—FIRST, Universidad Industrial de Santander, Bucaramanga 680002, Colombia; anamarial024@yahoo.com; 2 Grupo de Investigación en Recobro Mejorado—GRM, Universidad Industrial de Santander, Bucaramanga 680002, Colombia; proyecto.fluegas@uis.edu.co

**Keywords:** anionic surfactants, cationic surfactants, rheology, micellization, rheological enhancement, viscoelasticity, EOR

## Abstract

Enhanced oil recovery (EOR) methods traditionally rely on polymer solutions to improve viscosity and elasticity; however, their effectiveness is limited under high-temperature, high-salinity, and high-shear conditions, leading to elevated operational costs. Anionic/cationic formulations have been studied in terms of interfacial tension reduction for EOR applications. This study presents a novel approach to EOR by enhancing the rheological properties of an anionic internal olefin sulfonate surfactant through interactions with cationic surfactants, eliminating the need for polymer molecules. This research demonstrates that cationic surfactants can induce micellization changes, resulting in substantial viscosity enhancement and viscoelasticity development. The effect is found to depend on the hydrocarbon chain length and concentration of the cationic surfactants, with longer chains yielding higher viscosity and more pronounced non-Newtonian behavior. Additionally, this study reveals that the addition of NaCl alters micellar organization, with the order of component additions playing a critical role in rheological performance. This kinetic-dependent micellization behavior, rarely explored in EOR applications, highlights the potential of counterion surfactants as viscosity enhancers in surfactant-based flooding processes. Oscillatory rheology confirms that cationic/anionic surfactant systems in this study exhibit stable viscoelastic behavior, making them potentially more suitable for harsh reservoir environments than polymer-based EOR fluids. These findings open new avenues for the development of cost-effective and tailored surfactant formulations, offering an alternative to polymer solutions under challenging reservoir conditions.

## 1. Introduction

The injection of polymer solutions is widely employed to enhance oil recovery by displacing crude oil trapped by capillary forces in many oilfields worldwide. This technique is considered an enhanced oil recovery (EOR) method, offering several advantages but also facing certain limitations. When subjected to operating conditions, polymer solutions experience a reduction in their viscosifying effect, primarily due to high temperature, mechanical, chemical, and biological degradation, as well as high salinity [1]. These factors significantly influence viscosity and elasticity loss.

Another complex yet highly promising EOR approach involves the injection of surfactant solutions, which can achieve low or even ultra-low interfacial tension (IFT). This mechanism facilitates oil mobilization through droplet formation [2] and solubilization via the generation of microemulsions [3]. Moreover, the inclusion of surfactants in EOR formulations has been extensively reviewed, highlighting their role in improving oil displacement efficiency [4]. Additional studies on the emulsification and mobilization of paraffins using natural surfactants from fenugreek seeds have demonstrated their efficacy through pore-scale micromodel experiments [5]. Biosurfactants and natural surfactants have been studied for their ability to form stable emulsions and foams under saline conditions, making them viable candidates for alkaline-surfactant-polymer flooding applications [6,7]. These findings underscore the importance of surfactant formulation in enhancing oil recovery under various reservoir conditions [8,9]. Furthermore, the rheological characterization and salt tolerance of natural surfactant-based emulsions have been investigated for their potential in crude emulsification and mobilization [10].

The global oil recovery factor currently averages around 30% [11], though, in some cases, it can be as low as 10%. Improving recovery efficiency requires the implementation of advanced techniques, including thermal and chemical methods. The micellar/polymer flooding technique, which combines surfactants and polymeric molecules, has demonstrated significant potential in numerous studies. Surfactants contribute to IFT reduction, while polymer molecules enhance viscosity and elasticity, parameters that play a crucial role in displacement efficiency during flooding, as evidenced by recent research [12,13,14].

When implementing EOR strategies, oil companies seek not only to increase production and profitability but also to expand their recoverable crude oil reserves. Consequently, there is a growing need to develop innovative techniques that maximize hydrocarbon recovery while minimizing costs and ensuring environmental sustainability [15]. This is particularly critical in reservoirs where conventional polymers are ineffective due to extreme conditions such as high temperature and/or salinity.

Surfactants, characterized by their amphiphilic nature, contain both polar and non-polar segments, allowing them to stabilize or destabilize the oil–water interface by positioning their molecular structure accordingly [16]. As a result, they are typically found at the oil–water interface and are widely used across various sectors of the oil industry. The most significant properties of surfactant solutions include their ability to reduce surface and interfacial tension, enhance micellar solubilization, modify wettability, and generate foam [17]. When an optimized chemical formulation with an appropriate surfactant concentration is applied, high efficiency in oil recovery can be achieved [18]. However, in some cases, the formulation’s effectiveness is influenced by its viscosity, especially in heavy oil recovery. Unfavorable viscosities can lead to excessive chemical retention and suboptimal performance [19]. This issue cannot currently be addressed using surfactant molecules alone; instead, both natural and synthetic polymers have traditionally been employed to adjust the rheological behavior of these chemical formulations.

Surfactant formulations containing both anionic and cationic molecules have been previously tested for enhanced oil recovery, achieving minimal interfacial tensions. At the oil–water interface, these molecules attract each other [20], forming a composite molecular layer that reduces intermolecular repulsion [21]. This results in a more compact molecular arrangement and a more stable adsorption layer, thereby increasing the capillary number and improving oil mobilization in porous media [22]. The primary function of these anionic/cationic mixtures is to reduce interfacial tension rather than enhance viscosity and elasticity.

Recent studies have demonstrated that the viscosity (and potentially the elasticity) of anionic surfactant solutions can be significantly increased by the inclusion of inorganic salts, which induce substantial micellization changes [23]. The first efforts to enhance the rheological properties of surfactant solutions for EOR applications were conducted by Morvan et al. [24]. They concluded that viscoelasticity arises from the formation of wormlike micelles via surfactant self-assembly; however, this behavior is highly dependent on the formulation parameters, particularly salinity and temperature.

Subsequent research by Kamranfar and Jamialahmadi explored the effect of micelle-type transitions on the viscosity of a cationic surfactant formulation containing polymer molecules, induced by the addition of NaCl [25]. Their findings suggested that certain surfactants could simultaneously modify both IFT and viscosity, potentially eliminating the need for a polymer–surfactant mixture. The substitution of polymers in chemical formulations has also been investigated through the development of “polymeric surfactants”, as reviewed by Raffa et al. [26] and more recently by Afolabi et al. [27]. However, while polymeric surfactants can achieve the desired viscosity and elasticity, they do not always effectively reduce IFT.

In this context, counterion molecules, such as cationic surfactants, may play a crucial role in increasing the rheological behavior of organic compounds in aqueous solutions. When incorporated into chemical formulations, interactions with ionic surfactants can modify the rheological properties of the original anionic surfactant, by forming catanionic molecules, inducing micellar transitions, and enhancing viscosity and viscoelasticity.

Hodgdon and Kaler have extensively studied hydrotropes, particularly their strong synergistic effects when combined with surfactants or polymers [28]. The addition of oppositely charged hydrotropes to aqueous ionic surfactant solutions can alter critical micellar concentration, surface tension, viscosity, solubility, and microstructure, even at relatively low concentrations [25]. The influence of salts on surfactant microstructure has been widely studied. Recently, research on hydrotrope–surfactant mixtures has gained renewed interest due to the ability of hydrotropes to modify microstructural and microscopic properties. For instance, Hassan et al. [29] reported that hydrotropes can induce the transition of spherical micelles into elongated structures, significantly altering the solution’s rheological behavior.

The impact of salt type on physicochemical system properties was first observed over a century ago by Hofmeister, who investigated how inorganic electrolytes affect protein solubility. His findings indicated that anions exert a more substantial influence than cations [30]. Electrolytes also modify surfactant organization in water, either increasing or decreasing solubility (salting-in or salting-out effects, respectively). Given their influence on surfactant behavior, salts have become increasingly important in EOR applications. In particular, seawater, which contains approximately 3.5% wt sodium chloride (NaCl), has been used as a base fluid for surfactant solutions to induce viscoelasticity in chemical flooding systems [31].

The extensional rheological studies of surfactant–polymer mixtures have provided valuable insights into the behavior of these systems under flow conditions, highlighting their potential for EOR applications. Building on this foundation, an intriguing opportunity arises to further investigate the effects of cationic surfactants on an anionic surfactant solution within the context of EOR. This leads to the following research question: Can the viscosity curve and viscoelastic properties of an anionic surfactant formulation be enhanced by generating an anionic/cationic species that exhibits behavior comparable to traditional polymer-containing formulations? This study aims to enhance the rheological properties of a commercial anionic surfactant by incorporating a counterion cationic surfactant, with the goal of increasing viscosity and viscoelasticity in formulations that do not contain polymer molecules. Previous research on anionic/cationic surfactant mixtures primarily focused on reducing interfacial tension to mobilize trapped oil. In contrast, this study highlights the ability of cationic surfactants to enhance viscosity and viscoelasticity, providing an additional mechanism for mobility control. Additionally, it examines the impact of micellization changes induced by cationic species and the influence of salinity on rheological behavior, contributing to the development of polymer-free EOR formulations with enhanced performance under challenging reservoir conditions.

## 2. Materials and Methods

Materials: Anionic surfactants are commonly used in EOR applications due to their reduced adsorption onto typically anionic sandstone formations. For this study, a commercially available anionic surfactant widely used in EOR, Enordet O242 (EO242), was selected. Provided by Shell Chemicals (Houston, TX, USA), EO242 is an internal olefin sulfonate with a carbon chain length ranging from 20 to 24. The active material content was reported to be 20% (see Table 1).

Three cationic surfactants, supplied by Merck (Rahway, NJ, USA), were chosen based on their identical hydrophilic group (trimethyl ammonium bromide) but varying hydrocarbon chain lengths: dodecyl-trimethyl-ammonium bromide (12TMAB), tetradecyl-trimethyl-ammonium bromide (14TMAB), and hexadecyl-trimethyl-ammonium bromide (16TMAB). The purity of these compounds is detailed in Table 1. All aqueous solutions used in this study were prepared with ultrapure distilled water.

A partially hydrolyzed polyacrylamide, Flopaam^®^ 3430 (FP3430), manufactured by SNF Floeger (Andrézieux-Bouthéon, France), was used to prepare polymer solutions. This polymer has a reported molecular weight of 1.2 × 10⁷ Da. It is important to note that polymer solutions were prepared solely for comparison with anionic/cationic surfactant systems; therefore, surfactant solutions were formulated in the absence of polymer molecules. Additionally, sodium chloride (NaCl, purity > 99.8%), supplied by PanReac (Castellar del Vallès, Spain), was used as the inorganic salt.

Rheological measurements: Rheological data including viscosity curves and oscillatory measurements were collected using an Anton Paar MCR 302 rheometer (Graz, Austria), equipped with a CP50-1 measuring system. This is a 1-degree cone-plate geometry (50 mm diameter) for sample characterization (cone truncation of 101 μm). While oil reservoirs typically operate at temperatures exceeding 50 °C, this study was exploratory; thus, viscosity curves and oscillatory measurements were conducted at 25 °C. When operating under dynamic conditions, the shear rate was evaluated between 1 s^−1^ and 100 s^−1^. On the other hand, when operating under oscillatory conditions, shear strain was swept from 0.1 to 100% at a constant angular frequency of 10 rad/s. For frequency sweeps, all measurements were performed at a fixed strain (1%), ensuring working into the linear viscoelasticity region (frequencies from 0.01 to 100 rad/s).

All rheological measurements were conducted in triplicate, and the averaged results are reported in rheograms. Error bars were omitted, as variations were consistently below 5%, and their inclusion could hinder result visualization.

The EO242 surfactant concentrations were set at 1%, 3%, and 5% (*w*/*v*), while cationic surfactants concentrations were examined at 0.1%, 0.5%, 1%, 3%, and 5% (*w*/*v*).

Micelle size measurements: Micelle sizes in selected solutions were determined using dynamic light scattering (DLS) with a LiteSizer 500 instrument (Anton Paar, Graz, Austria). Measurements were performed at a scattering angle of 15°, maintaining a controlled temperature of (25.0 ± 0.1) °C.

## 3. Results and Discussions

### 3.1. Effect of Hydrocarbon Chain Length of the Cationic Surfactant on Rheological Behavior

**Figure 1:**
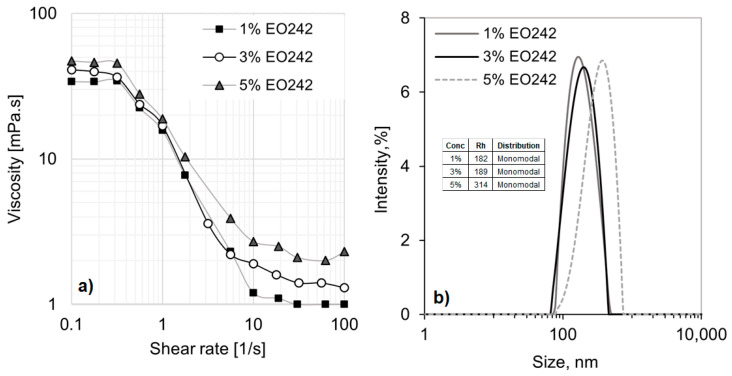
(**a**) Viscosity curves of EO242 solutions at several concentrations: (**b**) micelle size and distribution of EO242 at several concentrations.

The original anionic surfactant exhibits shear-thinning behavior at the concentrations used in this study. Figure 1a presents the viscosity curves for EO242 at various concentrations, revealing significant deviations from Newtonian behavior. This deviation is likely due to the formation of numerous micellar aggregates and the interactions among them.

At low shear rates, when the system is randomly dispersed and highly entangled, viscosity reaches 20 mPa·s at 5% EO242. However, as the shear flow aligns the microstructures (above 0.4 s^−1^), viscosity drops to minimal values, reaching 1 mPa·s at 1% surfactant concentration. This shear-thinning behavior is characteristic of concentrated nanostructured fluids [32], displaying two typical viscosity plateaus at low and high shear rates. Additionally, the plateau values at both low and high shear rates increase with higher surfactant concentrations. However, the differences are more pronounced at high shear rates, as shown in Figure 1a.

Meanwhile, only one type of micelle is formed at the concentrations used in this study, as indicated by the monomodal distributions observed in the DLS results (see Figure 1b). Moreover, the hydrodynamic radius (R_h_) of the micelles increases with anionic surfactant concentration, ranging from approximately 182 nm at 1% EO242 to 314 nm at 5% EO242. These results support the observed increase in viscosity with surfactant concentration, as the overall micelle population (and consequently, their self-interactions) becomes more pronounced at higher concentrations, even if the micelle size remains relatively consistent.

**Figure 2:**
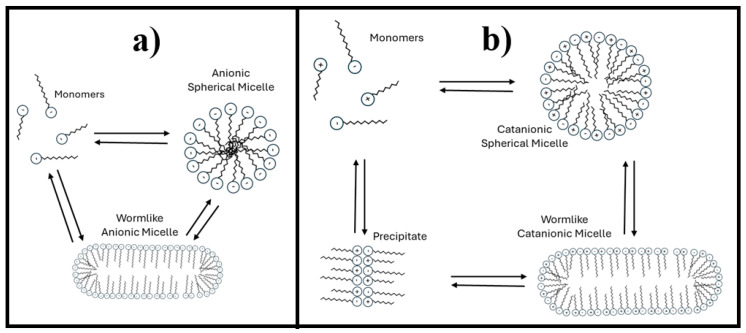
Likely micellization changes: (**a**) Anionic micelles. (**b**) Catanionic micelles.

The anionic micelles are likely worm-like, as suggested in Figure 2a. As the surfactant concentration increases, the size of these cylindrical micelles may grow, leading to a corresponding increase in the system’s hydrodynamic radius. Additionally, only a single type of micelle coexists, all of which are similar sizes.

Similarly, the rheological behavior of cationic surfactant solutions was evaluated. All solutions exhibited Newtonian behavior at concentrations of 1%, 3%, and even 5%, with viscosities comparable to that of water (~1 mPa·s).

**Figure 3:**
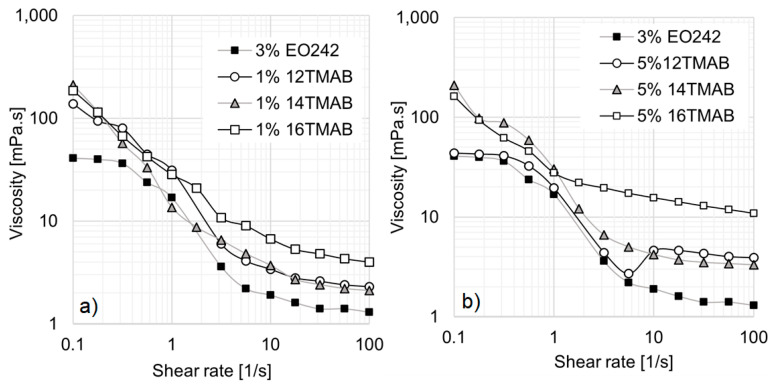
Viscosity curves of EO242 solutions at 3%: (**a**) Effect of cationic surfactants at 1%. (**b**) Effect of cationic surfactants at 5%.

For mixed systems containing EO242 and cationic surfactants, the salt concentrations were set at 0.1%, 0.5%, 1%, 3%, and 5% (*w*/*v*). However, selected systems were analyzed to evaluate the effect of hydrocarbon chain length on rheological behavior. Figure 3a presents the results for 3% EO242/1% cationic surfactant systems.

The first notable effect of incorporating cationic surfactants is the disappearance of the low-shear viscosity plateau in all solutions. Simultaneously, the hydrodynamic radius (R_h_) increases with the length of the cationic surfactant’s hydrocarbon chain. As shown in Table 2, a monomodal micelle distribution is observed for 12TMAB and 14TMAB, with R_h_ values of approximately 280 nm and 312 nm, respectively. However, in the 16TMAB system, a bimodal distribution emerges, suggesting the formation of two distinct micelle populations, with a significantly larger R_h_ of 1112 nm. In this study, there are limitations in determining the specific micellar transformations that occur. However, a potential micellar transition is proposed in Figure 2b. The system may transition from spherical micelles (associated with small hydrodynamic radius) to cylindrical micelles (characterized by a larger hydrodynamic radius) or a mixture of both types.

Referring again to Figure 3a, the 12TMAB and 14TMAB solutions exhibit a slight enhance in viscosity compared to the original EO242 system. However, the 16TMAB system demonstrates the highest viscosity values, particularly at shear rates above 1 s^−1^. The greater viscosity increase observed with longer alkyl chain lengths aligns with findings from Schulte et al. [33], who reported that micellar aggregates self-organize into supramolecular assemblies as a result of increasing activation energy and alkyl chain length at a given surfactant concentration. In other words, the co-surfactant’s chain length significantly influences the system’s rheological behavior, particularly its viscosity.

This effect becomes even more pronounced when the cationic surfactant concentration is increased to 5%, as shown in Figure 3b. At low shear rates, all systems exhibit similar behavior; however, at high shear rates, the 16TMAB solution reaches viscosity values up to ten times higher (10 mPa·s at 100 s^−1^).

The conclusions drawn by Schulte et al. can be further supplemented by considering that longer hydrocarbon chains enhance the hydrophobic character of the resulting surfactant. This occurs because a larger catanionic compound is formed, leading to the generation of larger micelles at lower concentrations. Consequently, as the carbon chain length increases, the number of micelles also increases, thereby intensifying micelle–micelle interactions and further elevating viscosity.

Additionally, DLS results (see Table 3) indicate the coexistence of two distinct micelle populations in these systems, as evidenced by bimodal distributions. At these concentrations, R_h_ also increases with the cationic surfactant’s hydrocarbon chain length, but all systems in this case exhibit two distinct micellar distributions. This behavior may be attributed to the coexistence of spherical and cylindrical catanionic micelles or the presence of spherical micelles with two distinct size distributions (see Figure 2b).

**Figure 4:**
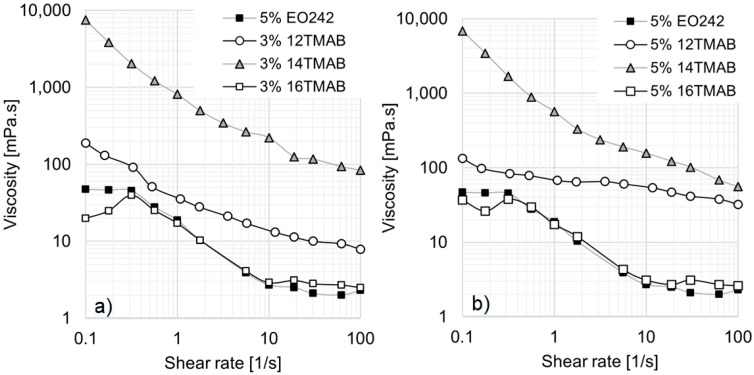
Viscosity curves of EO242 solutions at 5%. (**a**) Effect of cationic surfactants at 3%. (**b**) Effect of cationic surfactants at 5%.

It should be noted that not all systems studied in this work followed the expected trend, where a larger hydrocarbon chain size correlates with a higher viscosity profile. A notable exception is observed in systems containing 5% EO242 with 3% or 5% cationic surfactants (see Figure 4a,b). In these cases, as the EO242 concentration increases, a drop in viscosity is observed for 16TMAB systems, while the 12TMAB and 14TMAB systems exhibit a significant viscosity enhancement. This phenomenon may be attributed to surfactant solubility limitations at high concentrations (see Figure 2b), where any additional surfactant added no longer contributes to micelle formation [34]. In particular, the system containing 16 carbon atoms (16TMAB) appears to have lower solubility, despite having only two additional methylene groups. Once the system reaches saturation, further changes in viscosity are not observed, leading to a reduction in interactions between the anionic and cationic surfactants.

However, the behavior of the 14TMAB system is particularly noteworthy, as its viscosity reaches nearly 10,000 mPa·s at low shear rates, a hundredfold increase compared to the original EO242 system. These results align with findings from Schulte et al. [33], who reported that, above a certain cationic surfactant concentration, viscosity can increase by several orders of magnitude. At high shear rates, the viscosity increase remains significant, with no viscosity plateau observed for 12TMAB and 14TMAB systems within the studied shear rate range.

When comparing the cationic surfactant concentrations in Figure 4a,b, no substantial differences are observed for the 14TMAB and 16TMAB systems. However, the 12TMAB system exhibits a notable enhancement of viscosity at high shear rates, particularly when its concentration is raised to 5% (Figure 4b).

It is also essential to highlight the transition in micellar aggregates. At both 3% and 5% cationic surfactant concentrations, the system becomes bimodal for 12TMAB and 14TMAB (see Table 4 and Table 5). In these cases, the hydrodynamic radius (R_h_) increases with cationic surfactant chain length. Interestingly, the system reverts to a monomodal distribution for 16TMAB, with smaller micelles forming once again. This strongly suggests that micelle size and structure play a crucial role in determining the rheological behavior of these solutions.

### 3.2. Effect of Cationic Surfactant Concentration on the Rheological Behavior

Viscosity curves were obtained to determine the optimal cationic surfactant concentration at a fixed EO242 concentration. This section presents the systems that exhibited significant viscosity enhancement and notable deviations from Newtonian behavior.

**Figure 5:**
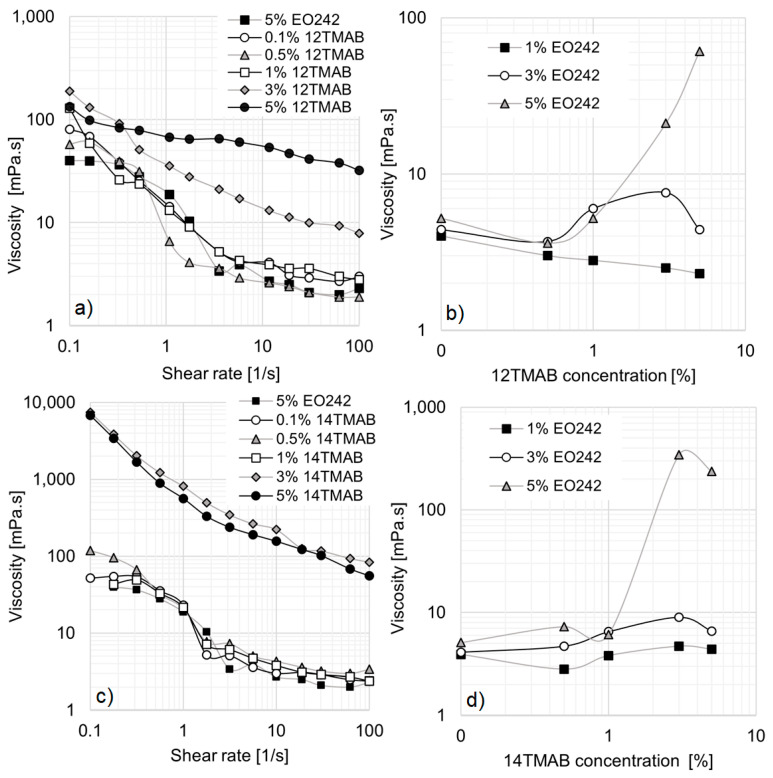
Effect of the cationic surfactants’ concentration on the viscosity of EO242 solutions at 5%: (**a**) Viscosity curves at several concentrations of 12TMAB. (**b**) Viscosity against the concentration of 12TMAB for various EO242 concentrations at 0.53 1/s. (**c**) Viscosity curves at several concentrations of 14TMAB. (**d**) Viscosity against the concentration of 14TMAB for various EO242 concentrations at 0.53 1/s.

Figure 5a illustrates the rheological behavior of 5% EO242 with varying 12TMAB concentrations. As previously discussed, the EO242 solution alone (without cationic surfactant) exhibits shear-thinning behavior, with zero-shear and high-shear plateaus at 39.9 mPa·s and 1.9 mPa·s, respectively. At low 12TMAB concentrations, no significant deviations from the original EO242 behavior are observed. However, when 12TMAB concentration exceeds 3%, viscosity enhances markedly, particularly at high shear rates. The zero-shear and high-shear plateaus disappear, and a higher viscosity profile emerges. This behavior suggests micelle aggregation into wormlike structures, which remain disordered at low shear rates, leading to higher viscosity due to increased intermolecular interactions. As shear rate increases, these structures align and reorganize, reducing viscosity, as demonstrated in other systems by Arenas-Gómez et al. However, despite the viscosity increase at 5% 12TMAB, the extent of shear thinning is reduced (Figure 5a).

Figure 5b shows the evolution of viscosity at a fixed shear rate (0.316 s^−1^) for the EO242-12TMAB system across all studied concentrations. At 1% EO242, viscosity initially decreases with increasing 12TMAB concentration, likely due to EO242 micelle disruption. In this scenario, the formation of catanionic molecules occurs, but their concentration is insufficient to generate new micellar aggregates, as the anionic surfactant limits their formation. Consequently, fewer EO242 micelles result in a decrease in viscosity at higher cationic surfactant concentrations.

A similar trend is observed at 3% EO242, where viscosity slightly decreases initially but begins to increase at 1% 12TMAB, likely due to the formation of micellar structures containing catanionic molecules. These structures appear to exceed a critical concentration, where their interactions become significant, leading to an increase in viscosity.

At 5% EO242, viscosity progressively increases from 0.5% 12TMAB onward, indicating a change in micellization. The original surfactant molecules likely combine with the cationic surfactant to form catanionic compounds, which enhance micelle interactions, ultimately resulting in a substantial viscosity enhance of up to 61.1 mPa·s. Additionally, a visible increase in turbidity is observed, though it remains unquantifiable due to all systems exceeding the upper detection limit of the equipment (1000 NTU).

Figure 5c illustrates the rheological behavior of the EO242/14TMAB system, showing a viscosity enhancement at 3% and 5% cationic surfactant concentrations. Under these conditions, the entire viscosity curve rises by approximately 170 times compared to the original EO242 system. These concentrated systems exhibit significant deviations from Newtonian behavior, with no observable zero-shear or high-shear plateaus within the studied shear rate range. This behavior is particularly relevant for EOR, where high viscosities at low shear rates improve mobility control and recovery efficiency, especially in heavy crude reservoirs [35].

The evolution of viscosity at a fixed shear rate (0.3 s^−1^) for EO242/14TMAB is presented in Figure 5d. No significant viscosity changes occur when 14TMAB concentration increases to 1% at 3% EO242, likely due to an insufficient concentration of catanionic micelles. However, in contrast to the EO242/12TMAB system, a slight viscosity increase is observed at 1% EO242, rather than a decline at 1% cationic surfactant. This may be attributed to enhanced interactions between larger catanionic molecules, as CMC decreases with increasing alkyl chain length.

When EO242 concentration is set to 5%, viscosity enhances significantly as 14TMAB concentration rises, reaching 343.8 mPa·s at 3% 14TMAB. This is likely due to the formation and growth of catanionic micelles, which, being larger and more flexible, can easily curve, leading to a rapid viscosity increase [29]. However, at 5% 14TMAB, viscosity ceases to increase, possibly due to a new micellization transition or solubility limitations. Turbidity changes are observable but unquantifiable, as values exceed the upper detection limit of standard turbidity meters.

**Figure 6:**
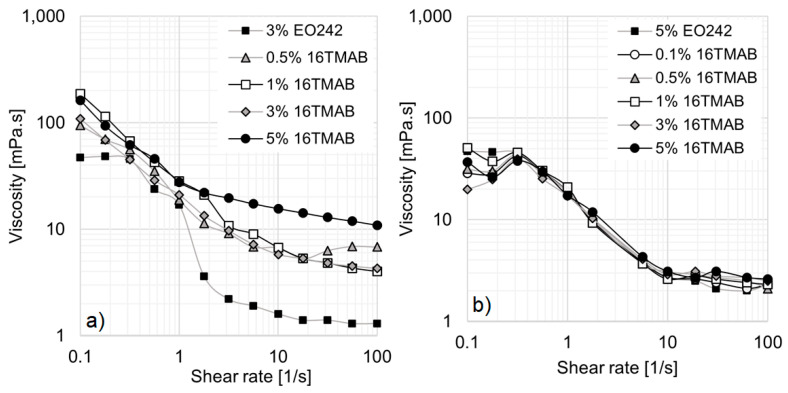
Effect of 16TMAB on the viscosity curve of EO242 solutions: (**a**) EO242 solutions at 3%. (**b**) EO242 solutions at 5%.

Focusing on the system with the longest hydrocarbon chain (16TMAB), Figure 6a,b show the viscosity evolution at 3% and 5% EO242, respectively. At 3% EO242, viscosity increases similarly to the 12TMAB and 14TMAB systems, likely due to catanionic molecule formation, which drives mixed catanionic/cationic micelle growth (as the cationic surfactant is present in excess relative to the anionic surfactant). The high-shear viscosity plateau rises with increasing 16TMAB concentration, reaching an order of magnitude increase (from 1.3 to 10.9 mPa·s) at 5% 16TMAB, indicating a strong dependence on salt concentration due to increased micellar interactions [23]. However, at 5% EO242, no significant viscosity changes occur with the 16TMAB addition (Figure 6b). This non-monotonic behavior is particularly intriguing, as 12TMAB and 14TMAB led to substantial viscosity increases (Figure 5), whereas 16TMAB appears to reach a solubility limit at high surfactant concentrations. The system exhibits notable turbidity, suggesting EO242 solubility limitations, which could explain the lack of viscosity enhancement. This inhibition of viscosity increase is also evident in Figure 4.

Up to this point, it can be concluded that viscosity increases moderately with a cationic surfactant addition at 3% EO242. However, at 5% EO242, the most significant viscosity improvement occurs with 12TMAB and 14TMAB, whereas 16TMAB does not lead to further viscosity enhancement, likely due to solubility limitations. In all cases, high turbidity is observed but remains unquantifiable as values exceed the detection limit of standard turbidity meters.

Based on these results, the systems exhibiting a notable viscosity enhancement and significant deviations from Newtonian behavior were selected for further analysis (see Table 6). This selection was primarily based on the theoretical viscoelastic properties exhibited by these systems, allowing for a comparison with the rheological viscoelastic properties of conventional hydrolyzed polyacrylamide (HPAM) solutions. In this context, high viscosity and pronounced non-Newtonian behavior can be indicative of viscoelasticity, which is a critical parameter for EOR applications. The ability of these systems to develop viscoelastic properties comparable to those of HPAM solutions underscores their potential for improving mobility control and oil displacement efficiency in EOR processes.

### 3.3. Oscillatory Characterization of the Most Promising Systems

**Figure 7:**
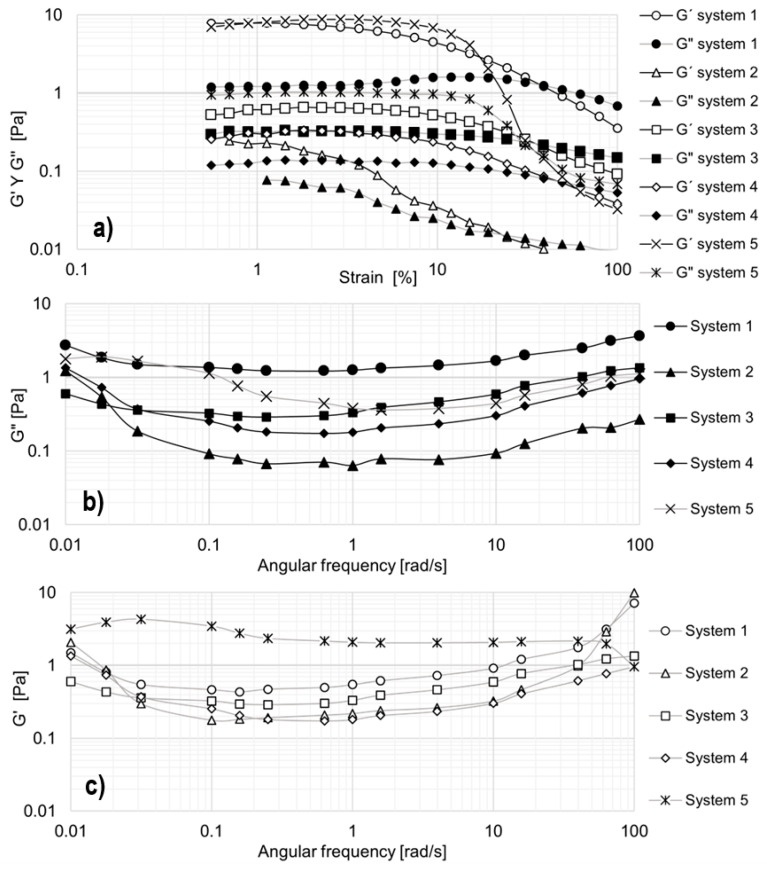
Oscillatory tests for most promising formulations: (**a**) G′ and G″ against strain. (**b**) Loss modulus against angular frequency. (**c**) Storage modulus against angular frequency.

To determine the linear viscoelastic region (LVR) of the selected formulations, strain sweep tests were conducted, measuring the storage modulus (G′) and loss modulus (G″) over a strain range of 0.1% to 100% (see Figure 7a). The angular frequency was fixed at 10 rad/s, and all measurements were carried out at 25 °C (see Figure 7b,c).

The 5% and 3% EO242 solutions were evaluated. However, in the strain sweep, the values of the elastic and viscous moduli are very low, indicating that these solutions exhibit minimal viscoelasticity. For instance, in the 5% solution, the elastic modulus ranges from 8 × 10^−2^ to 1 × 10^−3^ Pa, while the viscous modulus varies between 2 × 10^−2^ and 3 × 10^−3^ Pa. The 3% solution exhibits even lower values (G′ = [2 × 10^−2^ − 1 × 10^−3^] Pa; G″ = [8 × 10^−3^ − 6 × 10^−4^] Pa). In other words, although these solutions deviate from Newton’s law in terms of viscosity curves (Figure 1a), they ultimately do not exhibit elasticity. The results for these solutions are not presented, partly due to the high measurement errors and partly because they provide little additional insight beyond confirming the absence of viscoelasticity.

Different behaviors are exhibited by the formulations developed in terms of viscoelasticity. Some systems demonstrate the development of elasticity, as indicated by an increase in the storage modulus during oscillatory tests. By the way, the linear viscoelastic (LVE) region varies among formulations: up to 1% strain for system 2, 2% for systems 1 and 4, and 8% for systems 3 and 5. Within this range, all solutions exhibit elastic behavior (G′ > G″), meaning they respond instantaneously to stress and maintain a stable storage modulus (G′) as strain increases. Beyond the LVE region, G′ begins to decline, while G″ drops at higher strains, indicating a loss of elasticity due to micellar destabilization under high deformation. This suggests that micelles break, reducing intermolecular interactions [36]. The crossover point (G′ = G″) differs for each formulation, ranging from 24.3% strain (system 2) to 49.3% strain (system 4). These variations are likely due to differences in micelle flexibility or rigidity [37]. Notably, systems 1 and 5 exhibit the highest elasticity (see Figure 7a).

To further characterize the viscoelastic behavior, angular frequency sweeps were performed at 1% strain (within the LVE region) and 25 °C (see Figure 7b,c). In general, both moduli remain relatively constant across the studied frequency range. The storage modulus (G′) shows a slight decrease between 0.01 and 0.1 rad/s, except for system 5, where G′ decreases up to 1 rad/s, followed by a minor increase up to 100 rad/s. A similar trend is observed for the loss modulus (G″), which remains nearly constant with a slight rise from 0.1 to 100 rad/s. This behavior differs significantly from traditional polymeric solutions used in EOR, where both G′ and G″ increase by several orders of magnitude [38].

Comparing Figure 7b,c, it is evident that G′ remains consistently higher than G″, confirming that the systems exhibit dominant elastic behavior. This characteristic is advantageous for EOR applications, as maintaining viscoelasticity under different strain conditions enhances mobility control and recovery efficiency. Importantly, systems 1 and 5 demonstrated the highest viscoelasticity, which is particularly beneficial for medium-and low-permeability porous media in EOR processes [36].

### 3.4. Comparison of Formulations with Synthetic Polymer

**Figure 8:**
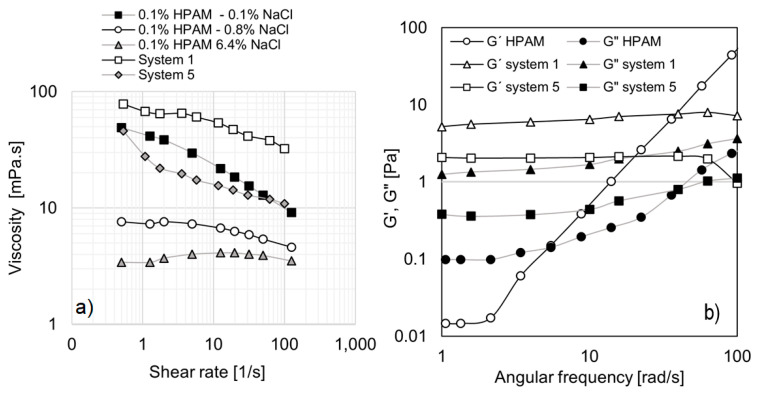
Rheological comparison of formulations with HPAM: (**a**) Viscosity curve against shear rate. (**b**) Storage and loss moduli against angular frequency at 1% strain.

For potential application of EOR, it is essential to compare the most promising formulations (systems 1 and 5) with a polymer traditionally used for the same purpose. In this study, a partially hydrolyzed polyacrylamide, HPAM-3430 (HPAM), was selected. The polymer solution was formulated to exhibit a viscosity curve comparable to those of systems 1 and 5. To achieve this, the polymer concentration was set at 0.1%, and its viscosity behavior was analyzed as a function of varying salinity levels (NaCl), as shown in Figure 8a.

The results indicate that increasing salinity leads to a reduction in polymer solution viscosity, which can be attributed to the screening of electrostatic repulsions, thereby weakening charge interactions [38]. Additionally, Figure 8a presents the viscosity curves of systems 1 and 5 for comparison. System 1 exhibits higher viscosities than HPAM at 0.1% NaCl, whereas system 5 generally shows lower viscosities. Given that the viscosity curves of these three systems are within the same order of magnitude, they were selected for further oscillatory tests to assess their rheological behavior.

Figure 8b presents the angular frequency sweep at 1% strain (within the linear viscoelastic (LVE) region) for system 1, system 5, and the selected polymer solution (0.1% HPAM at 0.1% NaCl). The behavior of HPAM is characteristic of an uncrosslinked polymer, particularly at low concentrations. In the lower frequency range, the loss modulus (G″) exceeds the storage modulus (G′), indicating a predominantly viscous response. However, at higher frequencies, both moduli increase, with G′ rising more rapidly than G″, leading to a crossover point at 5.47 rad/s. Beyond this point, the polymer exhibits significant elasticity. This behavior, which is characteristic of synthetic polymers, indicates that elasticity is dependent on high deformations within the porous medium. In other words, elasticity may manifest but is influenced by factors such as flow rate, pore size, and tortuosity.

In contrast, systems 1 and 5 behave similarly to crosslinked polymers, with G′ consistently exceeding G″ across the entire frequency range. Notably, both moduli remain nearly constant as frequency increases. For system 1, no crossover point is observed, while system 5 exhibits a sudden drop in G′ at 100 rad/s, which can be considered its crossover point. From this perspective, the micellar solutions identified in this study offer greater versatility, as they maintain elasticity across a broad range of deformations, ensuring this property under diverse flow conditions.

It is important to highlight that EO242/cationic surfactant systems maintain stable elastic properties over a wide range of frequencies. This characteristic could be advantageous for EOR applications, as these systems may retain elasticity under varying reservoir flow conditions. Fluids with higher elasticity generate significantly greater pressure drops during flow through porous media, which enhances microscopic sweep efficiency and improves oil recovery [39]. Unlike conventional hydrolyzed polyacrylamide (HPAM) solutions, which degrade in extreme conditions, the cationic/anionic surfactant systems in this study exhibit stable viscoelastic behavior, making them potentially more suitable for harsh reservoir environments.

The results aligned with expectations. However, achieving the desired effect requires very high concentrations of both anionic and cationic surfactants. Therefore, alternative systems are currently being investigated to reduce the required concentrations while ensuring a rheological response suitable for oil recovery processes.

### 3.5. Effect of NaCl on the Rheological Behavior of the EO242/16TMAB System

In EOR processes, injection water contains various inorganic salts, with NaCl as the most abundant component in brine. However, brine also includes divalent and trivalent cations, which significantly influence the viscoelasticity and viscosity of both synthetic and natural polymers [40]. While divalent and trivalent cations are known to affect polymer behavior, this study focuses on evaluating the impact of NaCl at different concentrations on the selected surfactant system.

The system chosen for this analysis was 3% EO242–5% TMAB (system 5, as defined in Table 2), as it demonstrated high viscoelasticity with the lowest surfactant concentration. Initially, its effect on viscosity curves was examined, with surfactant concentration kept constant while varying the NaCl concentration as the independent variable. Additionally, to assess whether the order of salt addition influences rheological properties, NaCl was incorporated using two different methods. The details of these preparation procedures are provided in Table 7.

**Figure 9:**
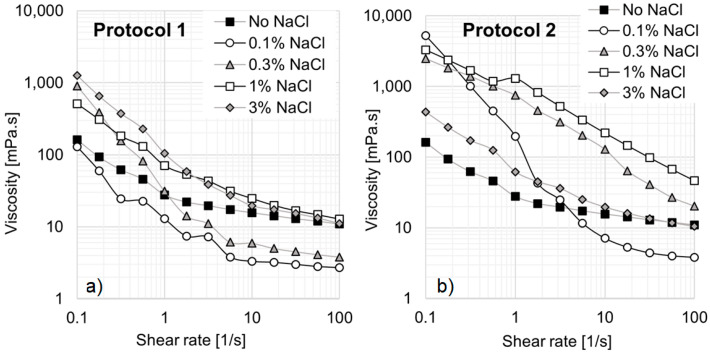
Effect of NaCl on viscosity curves of system 5: (**a**) According to Protocol 1. (**b**) According to Protocol 2.

In Figure 9, all solutions exhibit shear-thinning behavior, regardless of the preparation method used. However, significant differences arise depending on the order of the NaCl addition. Figure 9a presents results for solutions prepared according to Protocol 1, where no viscosity increase is observed at high shear rates. In contrast, at a shear rate of 0.1 s^−1^, the addition of NaCl leads to a substantial viscosity increase from 162 mPa·s to 1262 mPa·s. At a concentration of 0.1% NaCl, a noticeable change in rheological behavior occurs, as the viscosity decreases compared to the standard EO242/16TMAB solution without NaCl. Additionally, at 0.3% NaCl, the system deviates significantly from Newtonian behavior, despite the viscosity drop at high shear rates. When the salt concentration is further increased to 1% and 3% NaCl, the system exhibits a pronounced viscosity increase at low shear rates. This rise in viscosity can be attributed to the fact that, beyond a critical NaCl concentration, surfactant molecules interact more strongly, leading to the formation of larger micellar aggregates and structural changes in the micellar network, as demonstrated in previous studies [23,34].

Figure 9b presents the viscosity curves obtained following Protocol 2, which generally results in higher viscosities than Protocol 1. When the inorganic salt is added before the system, viscosity increases significantly from 162 mPa·s to 5250 mPa·s at 0.1 s^−1^. Notably, at 0.1% NaCl, the system exhibits a pronounced shear-thinning behavior, with viscosity reaching approximately 5250 mPa·s at low shear rates and decreasing to about 4 mPa·s at high shear rates. This behavior is particularly advantageous for enhanced oil recovery (EOR), as low viscosity facilitates pumping, while high viscosity enhances flow control in porous media, especially for heavy oil recovery. The system reaches its peak viscosity at 1% NaCl.

The addition of inorganic salt promotes hydrophobic interactions between surfactant molecules, which, beyond a critical concentration, become dominant over hydrophilic surfactant–water interactions, leading to spontaneous micelle formation [41]. In ionic surfactants, the reduction in the critical micelle concentration (CMC) is primarily attributed to the compression of the electrical double layer surrounding the micelles, which diminishes repulsive forces between hydrophilic groups and facilitates micelle formation at lower surfactant concentrations [42]. In other words, NaCl promotes viscosity enhancement by increasing micelle concentration. However, at 3% NaCl, viscosity decreases, likely due to micellization changes, suggesting that a maximum critical salt concentration exists beyond which viscosity declines.

One key observation is the influence of the order of compound addition. When the inorganic salt is introduced before the cationic surfactant (Protocol 2), the system exhibits generally higher viscosities. This effect may be due to the presence of a greater number of micelles at the time of cationic surfactant addition, leading to the formation of different micellar aggregates compared to when the cationic molecule is added first. This process facilitates a gradual micellar transition, where spherical, rod-shaped, or cylindrical micelles evolve into flexible worm-like micelles at lower inorganic salt concentrations [29].

As previously mentioned, the addition of salt influences the micellization of anionic surfactants. Specifically, electrolytes reduce the solvation of the surfactant’s hydrophilic region. Additionally, the presence of electrolytes increases the local ion concentration near the micelle surface, creating a shielding effect that diminishes electrostatic repulsions between the charged hydrophilic groups. Both effects promote micelle formation.

If salt is added to the anionic surfactant before introducing the cationic surfactant, a greater number of anionic micelles will form. As a result, the subsequent addition of the cationic surfactant may lead to a slower formation of catanionic micelles. Conversely, if salt is introduced into a system already containing both surfactants (anionic/cationic), it will enhance micelle formation but only after the catanionic micelles have already been established.

Results align with research conducted by Hassan et al. (2002) and Hodgdon and Kaler (2007), which explored how counterions alter micellization [28,29]. However, the findings go further by demonstrating that the order of salt addition impacts the final rheological behavior, a factor rarely explored in surfactant-based EOR.

**Figure 10:**
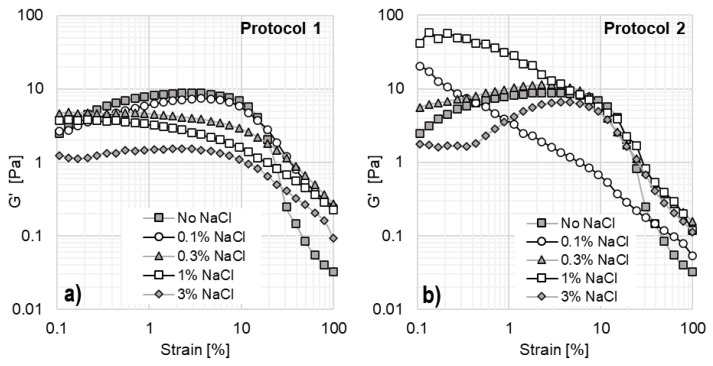
Effect of NaCl on the rheological properties of system 5 following two different protocols: (**a**) System prepared using Protocol 1. (**b**) System 5 prepared using Protocol 2.

To assess the impact of inorganic salt on viscoelastic properties, oscillatory tests were performed as a function of NaCl concentration and preparation protocol. The results are shown in Figure 10. Amplitude sweeps were conducted at 25 °C and a constant angular frequency of 10 rad/s, with only the elastic modulus (G′) reported as a function of shear strain.

For solutions prepared using Protocol 1 (Figure 10a), the elastic modulus is lower than that of the original system without NaCl. In contrast, for Protocol 2 (Figure 10b), elasticity does not follow a monotonic trend. Elasticity progressively increases with salt additions up to 1% NaCl but declines at 3% NaCl. At 0.1% NaCl, G′ continuously decreases with increasing strain, which aligns with the viscosity curve in Figure 9b. This suggests that no stable structures are formed at this concentration, as continuous micellization changes occur, with micelles breaking, reforming, and changing shape under strain. This behavior aligns with other studies since the electrostatic screening effect of salts can significantly influence micelle growth and viscoelasticity in surfactant solutions [43,44]. The addition of salt reduces electrostatic repulsions between charged surfactant head groups by compressing the electrical double layer around micelles. This lowers the critical micelle concentration and facilitates micelle formation at lower surfactant concentrations.

At 0.3% and 1% NaCl, the system exhibits higher elasticity compared to the EO242/16TMAB system without NaCl. The 0.3% NaCl solution forms stable structures at low shear strains (0.1–5%), but these structures begin to break down as strain increases. The 1% NaCl system exhibits the highest G′; however, it also shows a continuous decline in elasticity, similar to the 0.1% NaCl system. Finally, at 3% NaCl, elasticity is significantly reduced. In this case, G′ remains lower than in other systems and remains nearly constant between 0.1% and 0.4% strain, indicating the formation of stable micelles. However, as strain increases, G′ reaches a peak at 3.67% strain before continuously decreasing due to micelle breakage at high strain levels.

Significant differences in the rheological behavior of the systems are observed depending on the preparation protocol used. When solutions are prepared following Protocol 1, system elasticity decreases. In contrast, when prepared using Protocol 2, the storage modulus exhibits a nearly proportional increase with NaCl concentration (except at 0.1% NaCl). These findings indicate that the order of addition of inorganic salts and cationic surfactants leads to the formation of different micellar structures. As a result, NaCl can either enhance or reduce the elasticity of these systems, which are not at thermodynamic equilibrium, whereas traditional polymer solutions always reduce their elasticity with the addition of salt [45,46]. The slow kinetics of micellar formation is evident, as measurements taken after one week yielded the same results. This suggests that the kinetic pathways differ significantly between the two preparation protocols, making this an important factor to consider for potential applications in enhanced oil recovery (EOR).

## 4. Conclusions

This study establishes a relationship between the hydrocarbon chain length of the cationic surfactant and the viscosity behavior of the resulting catanionic/cationic micellar system. In general, as the chain length increases from 12 to 16 carbons, the viscosity profile also increases, though the behavior is not strictly monotonic.

Additionally, at low cationic surfactant concentrations, no significant increase in viscosity or elasticity is observed, regardless of the Enordet concentration. The effect of counterion surfactants on the rheological properties becomes more pronounced when both the Enordet and cationic surfactant concentrations exceed 3%. The increase in viscosity and elasticity is closely related to the shape, size, and concentration of the micellar aggregates formed. Furthermore, the observed viscoelastic properties of certain systems suggest that they may enhance the rheological behavior of anionic surfactant formulations even in the absence of polymer molecules.

The addition of NaCl can positively impact viscosity and elasticity in anionic/cationic surfactant systems. However, the order of component additions is a critical factor, as kinetic processes appear to be slow and influence the final rheological properties.

Despite these promising findings, the concentration of the anionic surfactant required to achieve substantial rheological changes remains high, posing economic challenges for field applications. However, this study serves as an initial exploration, opening the possibility of identifying alternative formulations that can significantly enhance rheology under realistic reservoir conditions. The prospects of this work involve developing formulations of anionic/cationic surfactant mixtures that meet the required specifications while exhibiting appropriate rheological behavior in terms of viscosity and elasticity. Additionally, these formulations can be tailored to the physicochemical conditions of the reservoir without the need for polymers, thereby offering enhanced stability. Consequently, future research should focus on optimizing surfactant systems that achieve these improvements while maintaining low interfacial tension and cost-effective concentrations suitable for industrial implementation.

## Figures and Tables

**Table 1:** Chemical compounds used in this study.

Compound	Name	Provider	Purity
Cationic surfactants	Dodecyl-trimethyl-ammonium bromide (12TMAB)	Merck	>98%
	Tetradecyl-trimetyl-ammonium bromide (14TMAB)	Merck	>98%
	Hexadecyl-trimethyl-ammonium bromide (16TMAB)	Merck	>98%
Anionic surfactant	Enordet O242 (EO242)	Shell	20%
Inorganic salt	NaCl	PanReac	>99%
Polymer	Flopaam^®^ 3430 (FP3430)	SNF Floeger	>99%

**Table 2:** Hydrodynamic radius and distribution of systems composed of 3% EO242 and 1% cationic surfactants.

System	R_h_, nm	Distribution
3%EO242	189	monomodal
1%12TMAB	280	monomodal
1%14TMAB	312	monomodal
1%16TMAB	1112	bimodal

**Table 3:** Hydrodynamic radius and distribution of systems composed of 3% EO242 and 5% cationic surfactants.

System	R_h_, nm	Distribution
3%EO242	189	monomodal
5%12TMAB	301	bimodal
5%14TMAB	606	bimodal
5%16TMAB	1533	bimodal

**Table 4:** Hydrodynamic radius and distribution of systems composed of 5% EO242 and 3% cationic surfactants.

System	R_h_, nm	Distribution
5%EO242	314	monomodal
3%12TMAB	766	bimodal
3%14TMAB	2240	bimodal
3%16TMAB	349	monomodal

**Table 5:** Hydrodynamic radius and distribution of systems composed of 5% EO242 and 5% cationic surfactants.

System	R_h_, nm	Distribution
5%EO242	314	monomodal
5%12TMAB	514	bimodal
5%14TMAB	1074	bimodal
5%16TMAB	317	monomodal

**Table 6:** Most promising solutions regarding potential viscoelasticity for EOR.

System	EO242 Concentration	Cationic Surfactant	Cationic Surfactant Concentration
1	5%	12TMAB	5%
2	3%	14TMAB	3%
3	5%	14TMAB	3%
4	5%	14TMAB	5%
5	3%	16TMAB	5%

**Table 7:** Preparation of the system following two different addition orders.

Addition Order	Protocol 1	Protocol 2
1	EO242 solution	EO242 solution
2	16TMAB solution	NaCl solution
3	NaCl solution	16TMAB solution
4	Water	Water

## Data Availability

The data presented in this study are available on request from the corresponding author due to privacy and legal restrictions.

## Abbreviations

The following abbreviations are used in this manuscript:

EOR	Enhanced oil recovery
EO242	Enordet O242
12TMAB	Dodecyl-trimethyl-ammonium bromide
14TMAB0	Tetradecyl-trimetyl-ammonium bromide
16TMAB	Hexadecyl-trimethyl-ammonium bromide
